# High-resolution microclimatic grids for the Bohemian Forest Ecosystem based on *in situ* measurements

**DOI:** 10.1038/s41597-026-06566-z

**Published:** 2026-01-14

**Authors:** Josef Brůna, Martin Macek, Matěj Man, Lucia Hederová, Tereza Klinerová, Vítězslav Moudrý, Marco Heurich, Jaroslav Červenka, Jan Wild, Martin Kopecký

**Affiliations:** 1 Institute of Botany of the Czech Academy of Sciences, 252 43 Průhonice, Czech Republic; 2 Faculty of Forestry, Technical University in Zvolen, Zvolen, 96001 Slovakia; 3 Department of Spatial Sciences, Faculty of Environmental Sciences, Czech University of Life Sciences Prague, Kamýcká 129, Praha, Suchdol 165 00 Czech Republic; 4 Faculty of Environment and Natural Resources, University of Freiburg, 79106 Freiburg, Germany; 5 Bavarian Forest National Park, 94481 Grafenau, Germany; 6 Institute for Forest and Wildlife Management, Campus Evenstad, Inland Norway University for Applied Science, NO-2480 Koppang, Norway; 7 Šumava National Park Administration, 385 01, Vimperk, Czech Republic

**Keywords:** Climate-change ecology, Forest ecology

## Abstract

High-resolution microclimatic temperature grids are needed for ecosystem modelling, biodiversity conservation, and forest management. However, existing climatic grids are coarse and do not capture microclimatic temperatures beneath tree canopies or near the ground. To address this, we established a dense network of microclimatic loggers continuously measuring air, near-ground, and soil temperatures. We combined one year of these *in situ* temperature measurements with high-resolution LiDAR-derived land surface topography and forest structure using boosted spatial generalized additive models to develop 5-m resolution microclimatic grids for the largest forest wilderness area in Central Europe, the Bohemian Forest Ecosystem (BFE). The resulting grids provide reasonable estimates of local annual maximum, mean, and minimum temperatures and growing degree days at different heights with spatially cross-validated RMSE values ranging from 0.41 °C for annual mean soil temperature to 2.34 °C for maximum near-ground temperature. Compared to SoilTemp (soil temperature), ForestTemp (near-ground temperature), and downscaled ERA5-Land (air temperature), the BFE microclimatic grids provide the most accurate local temperature estimates and capture substantially more spatial microclimatic variability.

## Background & Summary

Accurate climatic maps are crucial for environmental assessment, forest management, ecosystem modelling, and biodiversity conservation. However, standard climatic grids derived from weather station data do not capture microclimate at ecologically relevant scales^1,2^ and do not reflect microclimatic conditions in the forest understorey^3^. Forest microclimate is shaped by land surface topography and by canopy structure and composition^4–6^, creating a fine-scale microclimatic mosaic within the forest understorey that is absent from coarse-grained climatic grids^6,7^. Tree canopies buffer understorey microclimate from the free-air macroclimate measured by standard weather stations^8,9^. Moreover, the most buffered variables are the ecologically most important variables, maximum and minimum temperatures^10^. Although local weather stations can improve prediction of microclimate variation from global datasets^11^, reducing the discrepancy between the climatic conditions experienced by organisms (microclimate) and interpolated coarse-scale climatic variables (macroclimate) requires combining data from microclimatic measurement networks^12–15^ with spatially explicit data about land surface topography and forest structure to produce high-resolution microclimatic grids^2,13,16^. Such microclimatic grids stimulate ecological research^17^, improve species distribution modelling^18,19^, and facilitate forest management and nature conservation^20^.

**Fig. 1:**
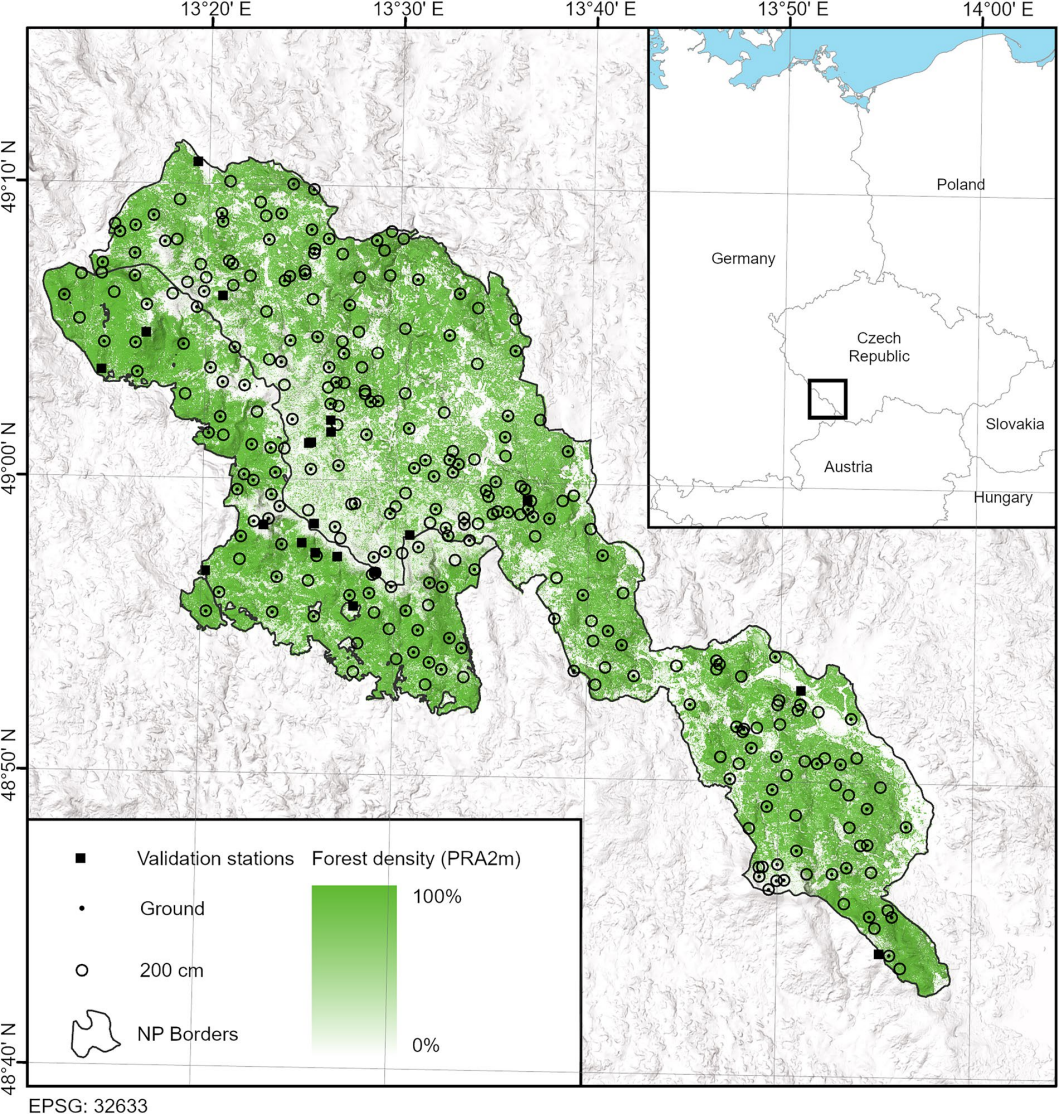
Microclimatic measurement network of the Bohemian Forest Ecosystem. Circles represent sites with air temperature measurements at 200 cm, dots represent sites with soil and near-ground temperature measurements, and squares represent the locations of independent weather stations used for model validation. Intensity of green colour represents forest density expressed as the proportion of LiDAR returns above 2 m (PRA2m). The underlying hillshade illustrates the topography based on the LiDAR digital terrain model.

To provide high-resolution microclimatic grids for the ecologically and biologically important Bohemian Forest Ecosystem (Fig. 1), we established a dense microclimatic network continuously measuring air, near-ground, and soil temperatures. We then combined these measurements with high-resolution LiDAR-derived predictors describing land surface topography and forest structure, and finally built spatial generalized additive models predicting microclimatic temperatures across 923 km^2^ of the Bohemian Forest Ecosystem at 5-m resolution. Here, we describe the data processing and model construction, assess the predictive performance of the resulting microclimatic grids with independent weather station data, and compare the microclimatic grids with the best available climate data sources.

The microclimatic grids of the Bohemian Forest Ecosystem provide mean annual air temperatures, as well as the biologically relevant minimum and maximum air temperatures^2,21,22^. Moreover, the grids provide temperature predictions at three different heights, allowing users to select the most relevant variable for the specific organism, environmental pattern, or natural process. Final models for each variable also included several predictors describing land surface topography and at least one predictor that describes forest structure. This illustrates that land surface topography and canopy structure should be included in forest microclimate modelling, especially when the modelled area includes recently disturbed stands with variable canopy density.

Microclimatic grids for the Bohemian Forest Ecosystem, an area with extensive prior study^23^, offer a wide range of potential applications. They can be used for high-resolution species distribution modelling^18,19^, in forest management aimed at maintaining favourable conditions for sensitive species^20^ and tree regeneration^24–26^, or for testing species distributional shifts^27^.

## Methods

### The study area

The Bohemian Forest Ecosystem is a 190 km long mountain range (561 to 1450 m a.s.l.) located on the border of Germany and the Czech Republic (Fig. 1). The German side of the mountain range falls steeply towards the Danube River, while the Czech side represents a flat high-elevation plateau interspersed with deeply incised valleys, glacial lakes, and extensive bogs and mires.

Areas above 1100 m a.s.l. are covered by Norway spruce (*Picea abies* (L.) H. Karst.) dominated forests, which experienced large-scale disturbances in the past decades, resulting in a current mosaic of forests with variable stand age, height, and density^28,29^. The lower elevations would naturally be covered by mixed montane forests of beech (*Fagus sylvatica* L.), fir (*Abies alba* Mill.), and spruce, but have been largely replaced by spruce plantations, especially on the northeast side of the mountain range.

This region constitutes the largest forest wilderness area in Central Europe and is protected by two National Parks (NPs): the Šumava National Park in the Czech Republic and the Bavarian Forest National Park in Germany.

### Sampling design

To collect microclimate data for model calibration, we established a permanent network of microclimatic loggers across the area of both National Parks in September 2019 (Fig. 1).

We based the network on existing forest inventory plots (n = 14 513) that cover the entire forested area in both National Parks in a regular grid with approximately 200 m spacing in the smaller Bavarian Forest NP and approximately 250 m spacing in the larger Šumava NP. To select sites for microclimate monitoring, we first excluded plots that experienced substantial canopy cover change (e.g. due to windthrow, logging, or bark beetle outbreaks) between 2017 and 2019 using vector data provided by both National Parks. We excluded these plots because the forest structure data were collected during the growing season of 2017.

Next, we classified the inventory plots into five elevation classes, three classes of potential direct solar radiation, and three classes of topographic wetness index using the Fisher-Jenks classification algorithm^30,31^ implemented in the classInt^32^ R package. The combination of these classes resulted in 45 strata. For each stratum present in Bavarian Forest NP, we randomly selected two plots for microclimate measurements.

In Šumava NP, where forests are generally more disturbed, we further divided each stratum into open and closed canopy stands using the median canopy cover (78%) value derived from tree canopy projections^33^ as a classification threshold. The combination of these classes resulted in 90 strata. From each stratum present within the Šumava NP area, we randomly selected two plots for microclimate measurements. Some strata combinations were not present in the area. To better represent the extremes of the elevation gradient, we added six plots at the highest and lowest elevations. This procedure resulted in the final selection of 270 plots covering the whole BFE area, where we established our microclimate measuring sites (Fig. 1). The resulting mean density is 0.3 sites per km^2^ within the study area. Coverage of environmental gradients in the study area vs. covered by our sites is in supplementary Fig. S10.

### Temperature measurements

**Fig. 2:**
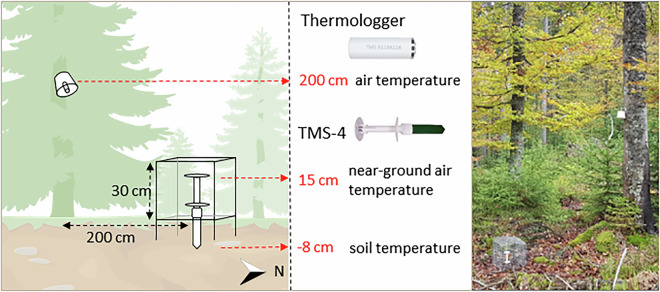
Design used for the microclimatic network of the Bohemian Forest Ecosystem. At each of the 270 sites, we measured the air temperature at 200 cm height with TOMST Thermologgers and at a subset of 150 sites and 18 independent sites, we also measured soil and near-ground temperature with TOMST TMS-4 loggers. The wire cages protect the TMS-4 logger against larger animals and are placed 200 cm from the nearest tree in the north direction. Drawing was created by Lucia Hederová, photograph by Josef Brůna.

At 270 sites, we measured the air temperature at 200 cm above the ground with TOMST Thermologgers attached to the north side of tree trunks and shielded by passively ventilated single-layer white plastic radiation shields (Fig. 2). At a subset of 150 sites, supplemented with 18 independent sites^34^, we also measured soil temperature 8 cm below the ground and the near-ground air temperature at 15 cm above the ground with TOMST TMS-4 loggers^35^. The TMS-4 loggers were placed 2 m north of the nearest tree, shielded with a standardised single-layer white plastic shield^35^ and were protected by cages against animal damage (Fig. 2). Both Thermologgers and TMS-4 loggers recorded the temperature every 15 minutes from October 12, 2019 to October 11, 2020. All loggers were equipped with the same MAXIM/DALLAS Semiconductor DS7505U + thermometers, which have an accuracy of ±0.5 °C and a resolution of 0.0625 °C. We recorded the geographic position of each site by differential GNSS with sub-metre positional precision after post-processing.

### Processing of microclimate data

After downloading the data, we visually inspected all temperature time series and removed data from problematic sensors. Because microclimatic temperature sensors can be systematically biased^36^, we therefore further adjusted the temperatures from each sensor using a fixed offset calculated as the average difference between the focal sensor measurements and the mean of simultaneous measurements performed by other sensors that were kept together in a controlled environment for at least 24 hours. These sensor-specific offsets were generally within the declared accuracy of the DS7505U + thermometer (0.5 °C) used in TMS-4 and Thermologgers. The mean offset for Thermologgers was 0.027 °C and ranged from −0.377 °C to 0.375 °C, for TMS-4, the mean offset was −0.015 and ranged from −0.336 °C to 0.313 °C.

Subsequently, we calculated nine variables summarising the annual microclimatic temperature of each site (Table 1) using the *myClim* R package^37^. We calculated the mean soil temperature from the TMS-4 soil sensor data at −8 cm. For air temperature, we calculated variables for two heights: near-ground air temperature (15 cm) from TMS-4 and air temperature (200 cm) from Thermologger. To capture different aspects of the thermal microclimate^14,21^, we calculated maximum temperature as the 95^th^ percentile of maximum daily temperatures and minimum temperature as the 5^th^ percentile of minimum daily temperatures for both the near-ground (15 cm) and the air (200 cm) temperature (Table 1). This definition of microclimatic extremes is robust to outliers, provides ecologically relevant estimates of temperature extremes, and is therefore often used in ecological studies^38^. From the air temperature measurements at 15 cm and 200 cm, we also calculated Growing Degree Days (GDD5) above 5 °C as the integral of all temperature measurements exceeding 5 °C divided by the number of measurements per day^37^.

**Table 1:** Microclimatic variables calculated from *in situ* temperature measurements performed in the permanent microclimatic network of the Bohemian Forest Ecosystem from October 12, 2019 to October 11, 2020. The methods used for the calculation of the microclimatic variables and their abbreviations follow the myClim R package^37^.

Temperature measurement (depth/height)	Logger/thermometer	Sites	Microclimatic variable		Abbreviation	Units
Soil temperature (−8 cm)	TMS-4 / DS7505U +	168	Mean temperature		T.soil_8_cm.mean	°C
Near-ground air temperature (15 cm)	TMS-4 / DS7505U +	168	Mean temperature		T.air_15_cm.mean	°C
			95^th^ percentile of daily maximum temperatures		T.air_15_cm.max.95p	°C
			5^th^ percentile of daily minimum temperatures		T.air_15_cm.min.5p	°C
			Growing degree days above 5 °C		T.air_15_cm.GDD5	°C d
Air temperature (200 cm)	Thermologger / DS7505U +	270	Mean temperature		T.air_200_cm.mean	°C
			95^th^ percentile of daily maximum temperatures		T.air_200_cm.max.95p	°C
			5^th^ percentile of daily minimum temperatures		T.air_200_cm.min.5p	°C
			Growing degree days above 5 °C		T.air_200_cm.GDD5	°C d

### Predictor variables

Within regions with similar macroclimate, forest microclimate is mostly shaped by land surface topography and forest structure^3^. Therefore, we used high-resolution LiDAR data to calculate two sets of predictors representing different aspects of land surface topography and forest structure with a potential effect on forest microclimate (Table 2). To quantify land surface topography and forest structure, we used airborne LiDAR data collected for the entire area of both National Parks using the Riegl LMSQ680i during the leaf-on period in June 2017^33^. After the post-processing by MILAN GeoService GmbH, the LiDAR point cloud had an average density of 55 points per m^2^. The digital terrain model (DTM) and digital surface model (DSM), each with 1-m resolution, were derived from the LiDAR point cloud by MILAN GeoService GmbH, referenced to DHDN/3-degree Gauss–Kruger zone 4 coordinate reference system (EPSG: 31468) and clipped to the area of both National Parks^33^.

**Table 2:** Predictors tested in the GAM models of microclimatic temperatures across the Bohemian Forest Ecosystem. All predictors were derived from LiDAR at a resolution of 5 m. Forest structure predictors and DAH were further spatially smoothed with the Gaussian filter. For the smoothed variables, the standard deviation of the selected filter is given in the Smooth column.

Variable (units)	Study area (min; max)		Study sites (min; max)	Smooth	Variable description
***Land surface topography***					
**DAH**	−0.89; 0.88		−0.41; 0.42	0, 10 m, 25 m	Diurnal anisotropic heating – approximation of incoming heat based on slope and aspect
**Elevation (m a.s.l)**	561; 1450		607; 1428		Elevation from LiDAR digital terrain model
**SAGAWI**	0;14.55		1.67; 11.12		SAGA Wetness Index – a TWI modification used mainly as a proxy for cold air pooling
**Slope (°)**	0; 79.90		0.34; 28.21		Slope of the terrain
**TPI_100**	−13.08; 18.75		−7.25; 7.07		Topographic Position Index (100 m radius)
**TPI_250**	−8.17; 10.02		−4.99; 6.66		Topographic Position Index (250 m radius)
**TPI_500**	−7.06; 6.91		−4.72; 6.38		Topographic Position Index (500 m radius)
**TWI**	0.71; 25.38		3.91; 15.18		Topographic Wetness Index – terrain-driven balance of the water supply from the upslope catchment area and local drainage driven by slope gradient
***Forest structure***					
**Conif_Cover (%)**	0; 100		0; 97	0, 10 m, 25 m	Proportion of coniferous trees in the canopy multiplied by the canopy density
**Decid_Cover (%)**	0; 100		0; 100	0, 10 m, 25 m	Proportion of deciduous trees in the canopy multiplied by the canopy density
**PRA2m (%)**	0; 100		0.00; 99.76	0, 10 m, 25 m	Canopy density – the proportion of LiDAR returns higher than 2 m above the ground
**Veg_Height (m)**	0; 46.26		0.20; 36.33	0, 10 m, 25 m	Vegetation height calculated as the difference between the digital surface model and the digital terrain model aggregated to 5 m resolution using the median value for each pixel

### Land surface topography

While 1-m resolution LiDAR DTMs capture land surface topography in extreme detail, many topography-driven processes influencing local microclimate operate at coarser scales^39^. Therefore, we resampled the 1-m LiDAR DTM to 5 m resolution using B-spline interpolation in SAGA GIS 7.9.0^40^.

To prevent edge artifacts in topographic variables, we supplemented the DTM covering only the area of both NPs with other LiDAR-based DTMs from Czechia (2 m resolution), Bavaria (10 m resolution) and Austria (1 arc-second resolution). Specifically, we first resampled these DTMs to 5 m resolution using B-spline interpolation and then merged them sequentially from high to low original resolution applying a distance-weighted average to the overlapping 25 m border zones. Through this process, we generated a final 5 m DTM covering the full area of both NPs as well as the neighbouring areas with potential influence on microclimate within the NPs.

Based on the previous literature^13,41,42^, we selected six topographic variables representing different processes affecting local microclimate (Table 2). As a principal land surface parameter affecting microclimate through the temperature lapse rate, we used elevation extracted from the 5 m DTM.

First, we calculated local slope with the second-order polynomial method^43^. Slope can affect microclimatic temperatures through its effect on air and water movement as well as the amount of received solar energy^44^.

Second, we calculated the Topographic Position Index (TPI), which expresses the difference between the elevation of a site and the mean elevation in its surroundings^45^. While exposed sites have positive TPI values, sites in topographic depressions have negative values. TPI therefore integrates local topographic effects on site exposure, cold air drainage, and moisture conditions and is often used in predicting fine-scale temperature patterns^46–48^. However, the TPI is strongly scale-dependent because its values depend on the area selected to compute the mean elevation^49^. Therefore, we calculated TPI with 100, 250, and 500 m radii and standardised TPI values for each radius by dividing the values by their observed standard deviations^49^.

Next, we calculated Topographic Wetness Index (TWI), which expresses the potential soil wetness as a terrain-driven balance of the water supply from the upslope catchment area and local drainage driven by slope gradient^50^. Sites with wetter soils have more buffered microclimatic temperatures^51^. Including TWI as a predictor therefore often improves microclimate modelling^13,16,52^. Here, we followed the TWI calculation guidelines^53^ and calculated TWI with the Freeman multiple-flow routing algorithm (flow convergence = 1.0), local slope^43^ and the flow width equal to the grid resolution^53^.

Although TWI is a useful proxy for soil moisture^53^, a TWI modification, called the SAGA Wetness Index (SAGAWI), was designed to better reflect cold air pooling in topographically flat terrain depressions^54^. Including SAGAWI often substantially improves microclimatic temperature predictions^2^. We calculated SAGAWI using the Freeman multiple-flow routing algorithm, local slope, and a suction factor of 10.

Finally, we calculated the Diurnal Anisotropic Heating (DAH) as a topographic proxy for site exposure to incoming solar radiation^54^. In contrast to solar radiation, which is symmetrical along the north-south axis, DAH sets the maximum heat surplus to 202.5° ^54^. DAH is therefore a more relevant proxy for microclimatic temperature patterns, because temperatures in the Northern Hemisphere are generally higher on the south-western slopes than on the south-eastern slopes^3^. To account for air mixing, which reduces the fine-scale differences in received radiation, we smoothed the DAH raster using a Gaussian filter with standard deviations of 10 m and 25 m. This smoothing has been shown to improve microclimate temperature modelling^2^.

### Forest structure

Forest structure affects microclimatic temperatures mostly through canopy height^8,55^, canopy density^56^, and tree species composition^57,58^. To account for these effects of forest structure on the understorey microclimate, we calculated several metrics from LiDAR (Table 2) using the lidR R package^59^.

First, we calculated the canopy height as the difference between DSM and DTM. As an estimate of canopy density, we used the Proportion of Returns Above 2 m (PRA2m) from all returns in the LiDAR point cloud. Finally, we calculated the proportional cover of deciduous and coniferous trees by combining the PRA2m metric and vector information on the tree type obtained from the single-tree crown delineation dataset^33^.

All forest structure variables were computed on the same 5-m grid as the topographic variables and were subsequently smoothed using Gaussian filters with standard deviations of 10 m and 25 m to account for the potential effects of a broader surrounding area on site temperature due to air mixing and interception of irradiance coming from lower zenith angles^3^.

### Microclimate modelling

We modelled microclimatic temperatures using spatial Generalised Additive Models (GAM) with automatic selection of predictor variables and their smoothing parameters based on gradient boosting implemented with the R packages geoGAM^60^, mgcv^61^, and mboost^62^. The principle of the method is to fit nonlinear relationships between predictors (in this case land surface topography and forest structure) and the dependent variable (*in situ* measured temperatures) using smooth terms (P-splines) that optimise the complexity of the smooth term to achieve the best predictive ability (i.e. minimise the mean deviation of the predicted values). To account for the potential autocorrelation of model residuals and for potential broad-scale macroclimatic patterns not reflected by our fine-scale topographic and structural variables, we used a spatial tensor, calculated from geographic coordinates, as a predictor in all models^61^. To account for possible interactions between topographic heat load and canopy density approximating solar radiation reaching the forest floor, we tested the addition of an interaction tensor product between DAH and PRA2m. Because this interaction term cannot be defined within geoGAM, we added this term to the final GAM model, and we used the resulting change in cross-validated root mean square error (RMSE) as a criterion for including the interaction term.

To assess the performance of the final model for each microclimatic variable, we used a ten-fold spatial cross-validation. We partitioned the study area into a regular grid of hexagons (≈9 km flat-to-flat) and assigned each microclimate site to its corresponding hexagon. Hexagons were then allocated to one of ten folds such that each fold contained multiple hexagons and the total number of microclimate sites per fold was approximately balanced. The GAM model was iteratively fitted to the data from nine folds and the microclimatic sites from the remaining fold were used to calculate the model RMSE. This procedure was repeated ten times, each time calculating RMSE for microclimate sites in a different spatial fold. To obtain coherent results, we used the same folds for evaluating models for all microclimatic variables. Finally, we calculated the mean RMSE for each modelled variable as an average of ten individual RMSE.

Additionally, we checked whether the shape of the partial relationships between the predictor variables and the microclimatic variables is consistent with the physical principles. We also explored the model predictions beyond the data range used for the model training (i.e. model extrapolation). To visualise the areas where BFE microclimatic grids extrapolate beyond the range of predictor variables covered by our training data, we prepared an uncertainty raster that quantifies for each pixel the proportion of variables used in the model that are beyond the variable range covered by the training data (Fig. 7).

Using the final GAM model for each microclimatic variable (Table 3), we generated the BFE microclimatic grids in GeoTIFF format by applying the model to the entire area of the Bohemian Forest Ecosystem.

**Table 3:** Summary of spatial GAM models predicting microclimatic temperatures over the Bohemian Forest Ecosystem. Table shows the smoothing coefficient for each predictor variable entering the model, the variation explained by the model (adjusted R2), and the root mean square error (RMSE) of the model calculated as the average RMSE from ten-fold spatial cross-validation. Smoothing coefficient = 1.00 means a linear relationship, higher values denote more complex non-linear response. Each value includes a sign describing the general shape of the relationship: ↑ positive effect, ↓ negative effect, ∩ unimodal, ∪ inverse unimodal (detailed graphs are in Fig. S01-S09). The predictor variables (except the spatial tensor) are sorted according to the decreasing number of models in which they were selected to the final model for a given microclimatic variable, which can be used as an indication of the variable importance. Names of predictors with suffix _g10 or _g25 denote the standard deviation used for smoothing.

Modelled variable	Selected predictors															Model performance	
	Elevation	DAH_g25	SAGAWI	Veg_Height	PRA2m_g25	Veg_Height_g25	DAH	TPI_500	TWI	Decid_Cover_g25	PRA2m_g10	TPI_100	Slope	Conif_Cover_g10	Spatial Tensor (longitude * latitude)	Adjusted R2	RMSE
**T.soil_8_cm.mean**	2.14 ↓		1.25 ↓	3.86 ∪			1.00 ↑		1.00 ↑						10.72	0.73	0.41
**T.air_15_cm.mean**	1.00 ↓	3.47 ∪	2.02 ↓			3.98 ∪									8.84	0.79	0.46
**T.air_15_cm.max.95p**	1.00 ↓			1.00 ↑	1.70 ↓		1.00 ↑									0.56	2.34
**T.air_15_cm.min.5p**	3.00 ↑				3.28 ∪										3.00	0.49	1.12
**T.air_15_cm.GDD5**	1.00 ↓	2.67 ∪	2.05 ↓	3.95 ∪											7.46	0.67	129.07
**T.air_200_cm.mean**	3.61 ↓	2.34 ↑	2.19 ↓			4.16 ∪				1.89 ↑					10.60	0.76	0.42
**T.air_200_cm.max.95p**	2.83 ↓	1.00		2.03 ↑	3.04 ∩						1.00 ↓	1.00 ↑			4.29	0.7	0.99
**T.air_200_cm.min.5p**	4.87 ↓				1.00 ↑	1.00 ↑		3.60 ∪	4.48 ∪				3.42 ∩		4.43	0.72	0.97
**T.air_200_cm.GDD5**	4.17 ↓	2.45 ↑	1.68 ↓	3.25				1.36 ↑						1.00 ↓	11.04	0.79	87.87

### Software used

For processing geospatial data, data analyses, and visualisations, we used SAGA GIS^40^ and R^63^ supplemented with the already cited R packages and the R packages terra^64^, rgdal^65^, raster^66^, sf^67^, and ggplot2^68^. To ensure visual accuracy and consistency, all maps were generated using scientific colour maps^69^ in ArcGIS Pro^70^.

## Data Records

High-resolution microclimatic grids for the Bohemian Forest Ecosystem are freely available in the Zenodo repository: https://zenodo.org/records/17671120 with 10.5281/zenodo.17671120^71^. The dataset contains nine microclimatic grids (GeoTIFF format, EPSG 31468, resolution 5 m). The file names represent the abbreviation of the microclimatic variable (Table 1). The data can be readily imported into geographic information systems (e.g., QGIS) or statistical software (e.g., R).

The repository also includes code and data used for modelling “BFE_microclimate_maps_model_script_final.R” and an RData file with values of microclimate variables and predictor variables for all stations (BFE_data2.RData). It includes the dataframe “zeta” with 62 variables for all 288 plots, SpatialPointsDataFrame “zeta.sp” with the spatial data of all plots and their unique ID “ID_lokalit” and a simple list with the names of predictor variables, “stack_names”. The repository also includes “microclimate2predict.csv”, which was used to select variables for modelling and includes full names and units.

## Technical Validation

To validate the BFE grids, we used four approaches: we 1) examined the model accuracy through spatial cross-validation as described in methods, 2) assessed the model predictions with independent climate stations, 3) compared the BFE grids with the best currently available gridded products, and 4) compared our measured temperatures with values of the best available gridded products.

### Microclimatic modelling accuracy

**Fig. 3:**
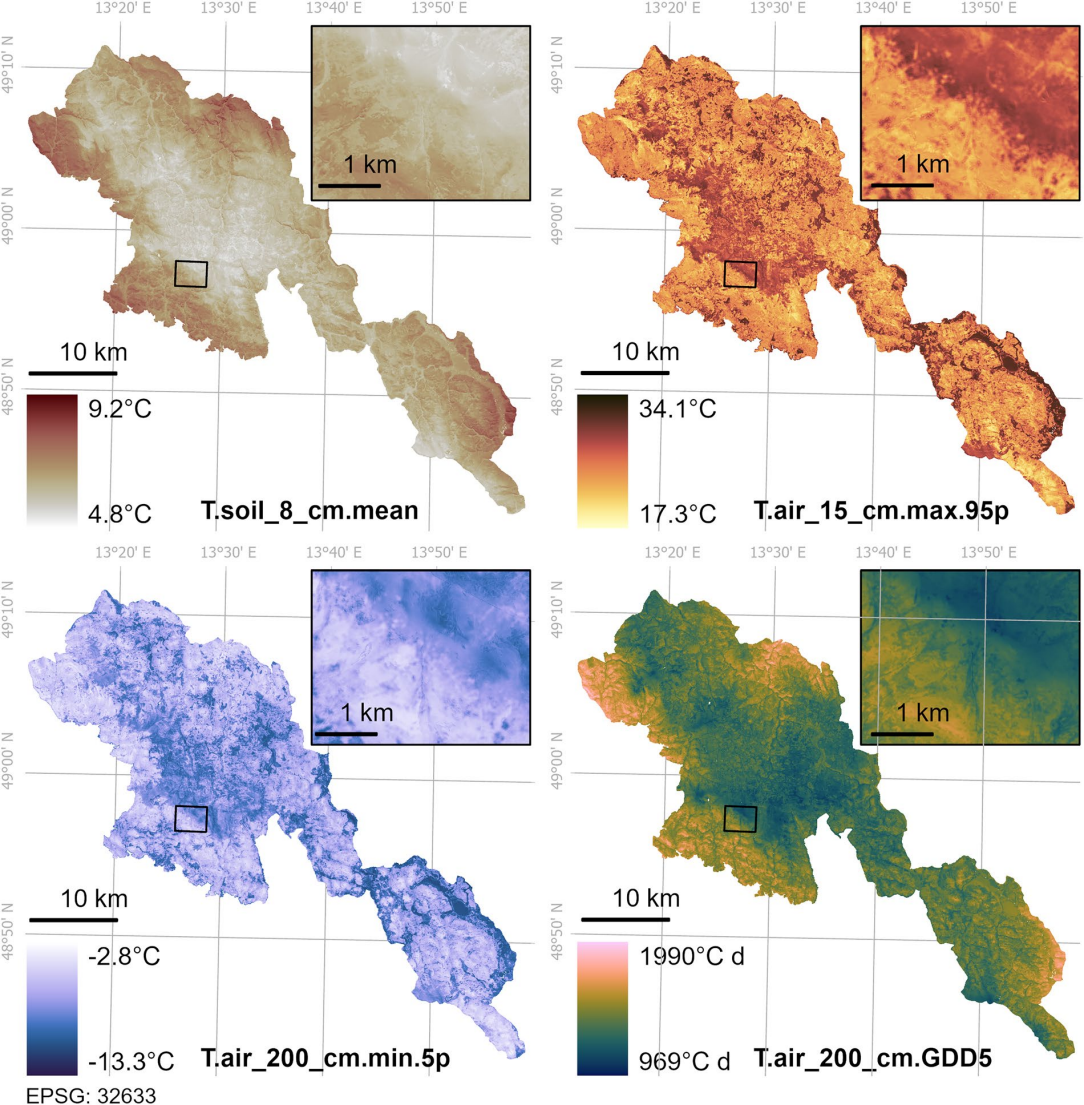
Examples of microclimatic grids for the Bohemian Forest Ecosystem. Each grid has a spatial resolution of 5 m and covers the entire area of both National Parks (Šumava in the Czech Republic and the Bavarian Forest in Germany). The inset maps illustrate fine-scale microclimatic variability around Mount Lusen. The numbers next to each colour map represent the range of each modelled temperature variable within the Bohemian Forest Ecosystem.

We modelled all microclimatic variables with reasonable accuracy (Table 3). Adjusted R^2^ values of the models ranged from 0.49 to 0.79, depending on the modelled variable (Table 3). Based on ten-fold spatial cross-validation, theRMSE ranged from 0.41 °C to 2.34 °C for microclimatic temperatures, and the RMSE of GDD was 87.87 °C d for 200 cm and 129.07 °C d for 15 cm (Table 3). The microclimatic grids based on these models revealed a high spatial variability in the microclimatic conditions across the Bohemian Forest Ecosystem (Fig. 3).

The models predicted mean temperatures and growing degree days most accurately, whereas the predictions for temperature extremes were less accurate (Table 3). Predictions were also substantially more accurate for air temperatures at 200 cm than for near-ground temperatures (Table 3).

Elevation was the most frequently selected predictor in all models (Table 3). The next most frequently selected predictor was diurnal anisotropic heating. Each final model also included at least one predictor that described forest structure, although the selected predictors often differed among models (Table 3). The vegetation height was more often selected as a predictor of mean temperatures and growing degree days, while the canopy density (PRA2m) was consistently selected in the models of temperature extremes.

### Validation of microclimatic grids

We validated the BFE microclimatic grids using an independent set of 19 forest weather stations measuring air temperatures at 200 cm every 10 minutes (Fig. 1). First, we calculated the same set of microclimate variables (Table 1) from the weather station data for the period of our microclimatic measurements. Then, we compared these independently calculated variables with the corresponding variables extracted from BFE microclimatic grids at the weather station coordinates.

The developed BFE microclimatic grids were in good agreement with the air temperatures measured at independent weather stations (Table 4). The modelled annual mean temperature at 200 cm was generally slightly lower than the observed values (mean error = −0.23 °C), but reasonably accurate (RMSE = 0.65 °C). The 5^th^ percentile of minimum daily temperatures was modelled almost without bias and with reasonable accuracy (mean error = −0.04 °C, RMSE = 1.56 °C), while the modelled 95^th^ percentile of maximum daily temperatures was generally slightly underestimated (mean error = −0.48 °C, RMSE = 2.17 °C). Growing degree days were also slightly underestimated (mean error = −34.85 °C d, RMSE = 126.51 °C d).

**Table 4:** Differences between air temperature variables calculated from measurements performed at 200 cm at 19 independent weather stations and the same variables extracted from BFE microclimatic grids, downscaled ERA5-Land grids, and ERA5-Land grids, expressed as mean error (ME), mean absolute error (MAE), and RMSE. ME, MAE, and RMSE were calculated as grid minus station. For a better comparison with the downscaled ERA5-Land, we also calculated the zonal mean of the BFE microclimatic grid at the same resolution as the downscaled ERA5-Land. The lowest error for each temperature variable is typeset in bold.

Variable (unit)	Data source	Resolution	ME	MAE	RMSE
T.air_200_cm.mean (°C)	BFE microclimatic grid	5 m	−0.23	**0.49**	**0.65**
	Zonal mean of BFE grid	~740 m	**−0.12**	0.51	0.69
	Downscaled ERA5-Land	~740 m	1.13	1.2	1.35
	ERA5-Land	~9 000 m	1.55	1.56	1.79
T.air_200_cm.min.5p (°C)	BFE microclimatic grid	5 m	**−0.04**	**1.28**	**1.56**
	Zonal mean of BFE grid	~740 m	0.72	1.29	1.77
	Downscaled ERA5-Land	~740 m	2.78	2.9	3.76
	ERA5-Land	~9 000 m	3.42	3.44	4.09
T.air_200_cm.max.95p (°C)	BFE microclimatic grid	5 m	**−0.57**	1.63	2.20
	Zonal mean of BFE grid	~740 m	−1.1	**1.43**	**1.86**
	Downscaled ERA5-Land	~740 m	−1.85	1.99	2.43
	ERA5-Land	~9 000 m	−1.23	1.93	2.35
T.air_200_cm.GDD5 (°C d)	BFE microclimatic grid	5 m	**−41.92**	**94.78**	**131.73**
	Zonal mean of BFE grid	~740 m	−42.99	111.38	149.24
	Downscaled ERA5-Land	~740 m	214.69	224.01	251.87
	ERA5-Land	~9 000 m	307.71	312.77	360.01

To put the results from the BFE microclimatic grids validation into perspective, we compared the temperatures from independent weather stations to the ERA5-Land climate reanalysis. ERA5-Land represents state-of-the-art in climate modelling and provides hourly meteorological data at 9 km resolution. We compared temperatures from the 19 independent weather stations to ERA5-Land temperatures both at the original ERA5-Land resolution (9 km) and at the finer resolution of ERA5-Land downscaled to 740 m using the KrigR R package^72^.

Compared to the downscaled ERA5-Land, the values extracted from BFE microclimatic grids were substantially closer to the temperatures measured at independent weather stations, both at the original 5 m resolution and at the resolution coarsened to match the resolution of the downscaled ERA5-Land (Table 4). Therefore, BFE microclimatic grids provide substantially more accurate estimates of air temperature, even when aggregated to a coarser resolution.

### Comparison with other climatic grids

#### Soil temperature

We compared mean annual soil temperature from the BFE microclimatic grids with Global maps of soil temperature (SoilTemp), version 2^73^. SoilTemp grids provide global estimates of soil temperature at 30 arc seconds resolution for two soil depths (0–5 cm and 5–15 cm). Here, we used the SoilTemp annual mean temperature grid for 5–15 cm, which is largely based on measurements from TMS-4 loggers and is therefore most comparable to our BFE microclimatic grids. For comparison, we also aggregated the BFE high-resolution grids of soil temperature to the 30 arc seconds resolution of SoilTemp using zonal means. To summarise the differences, we plotted the density distribution of the differences at both resolutions.

**Fig. 4:**
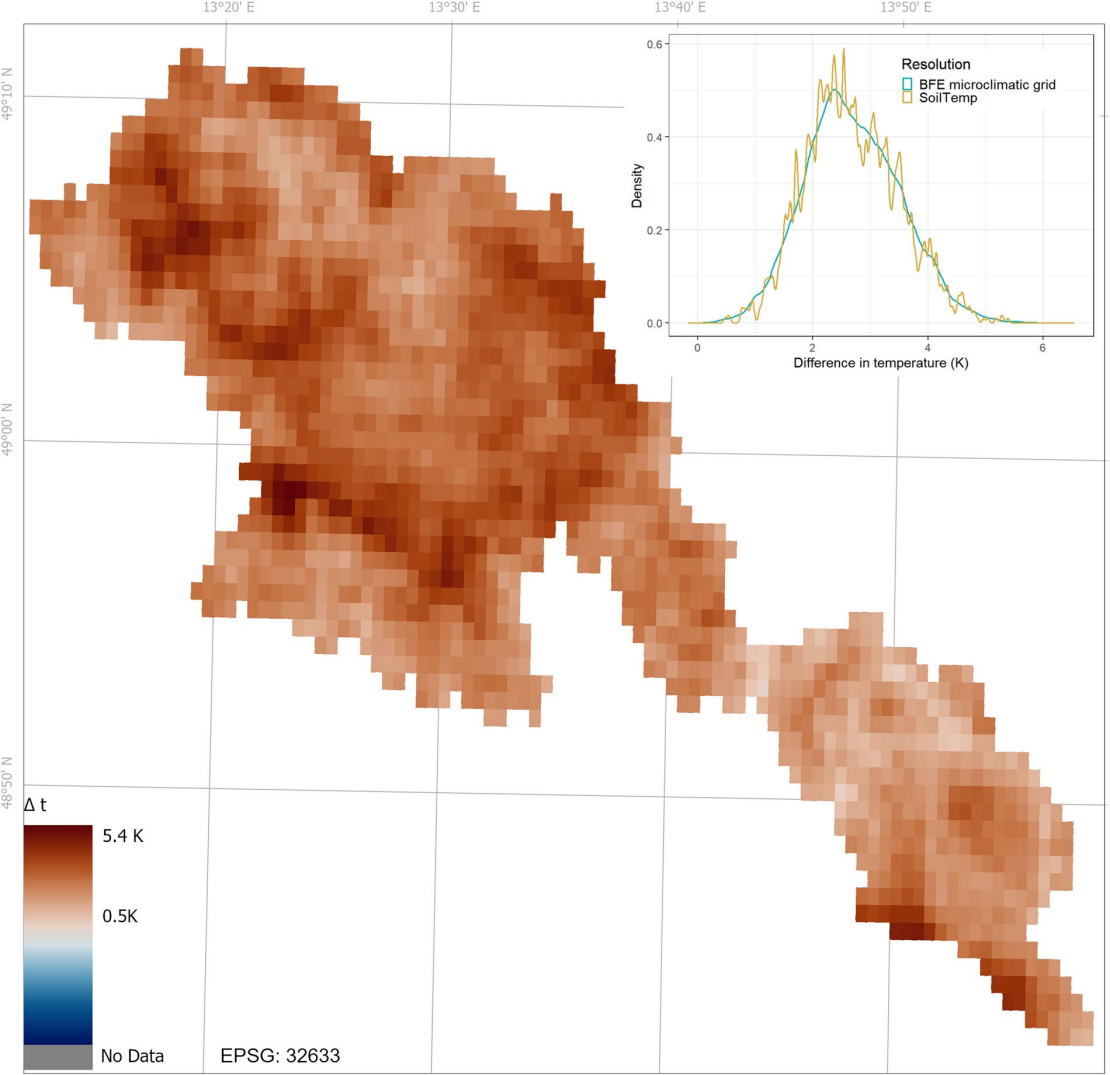
The difference in annual mean soil temperature between the microclimatic grids for the Bohemian Forest Ecosystem and SoilTemp (BFE - SoilTemp). The map shows the spatial patterns of temperature differences at the SoilTemp resolution (~1 km), and the density plot shows the distribution of the differences at both the 5 m resolution of the microclimatic grids and at the SoilTemp resolution. The numbers next to the zero-centred colour map represent the range of soil temperature differences between the zonal means of the BFE microclimatic grids and SoilTemp observed within the Bohemian Forest Ecosystem. Positive values indicate BFE warmer than SoilTemp.

The BFE microclimatic grids predicted substantially warmer soil temperatures and a narrower range of values than SoilTemp in the study region (Fig. 4). The mean difference between the BFE mean annual soil temperature grid aggregated to the SoilTemp resolution and the SoilTemp mean annual temperature grid was 2.68 °C, indicating that BFE grids are on average 2.68 °C warmer than SoilTemp in the study area (Table 5). The SoilTemp grid clipped to the Bohemian Forest Ecosystem area has a range from 0.42 °C to 6.25 °C, while the BFE microclimatic grid aggregated to the same resolution had generally warmer temperatures, but with narrower range from 5.41 °C to 8.06 °C (the BFE microclimatic grid in the original 5 m resolution had a range from 4.84 °C to 9.19 °C). Despite this narrower range at 1 km resolution, the 5 m BFE grids still capture strong fine-scale spatial variation in soil temperature.

**Table 5:** Comparison of respective zonal means of BFE grids with available products. ME, MAE, and RMSE were calculated as BFE zonal mean minus external product.

Variable (unit)	Data source	Observations	Resolution	ME	MAE	RMSE
T.soil_8_cm.mean (°C)	SoilTemp	1688	1000 m	2.68	2.68	2.79
T.air_15_cm.mean (°C)	ForestTemp	1 190 245	25 m	0.05	0.43	0.54
T.air_200_cm.mean (°C)	Downscaled ERA5-Land	1879	740 m	−1.38	1.38	1.45
T.air_200_cm.mean (°C)	ERA5-Land	29	9 000 m	−1.59	1.59	1.68

The greatest differences were observed in areas with disturbed forests, where SoilTemp predicted a substantially lower mean annual temperature, in some cases up to 5.4 °C lower than the BFE grids (Fig. 4). These differences were even more pronounced at the original 5 m resolution (difference of up to 6.54 °C). The geographic distribution of these differences was not random (Fig. 4). The differences were smaller at lower elevations and in closed canopy forests, while higher differences were observed in recently disturbed forests and at higher elevations. Since the SoilTemp grid was calculated from a longer time series that included substantially older data, some of these absolute differences may also be explained by the ongoing climate change.

#### Near-ground air temperature

We compared the mean annual near-ground air temperature from the BFE microclimatic grids with ForestTemp grids, which provide 25 m resolution maps of mean annual near-ground air temperatures in European forests^7^. We used the corrected and updated version of the ForestTemp grid published in September 2022^74^. ForestTemp covers forests as defined by the Copernicus 2015 high-resolution (20 m) tree cover density map. Due to recent large-scale disturbances in our study area, many forested areas were classified as non-forest in the Copernicus product, resulting in ForestTemp not covering 36 km^2^ (4.6%) of forest habitats in the study area.

**Fig. 5:**
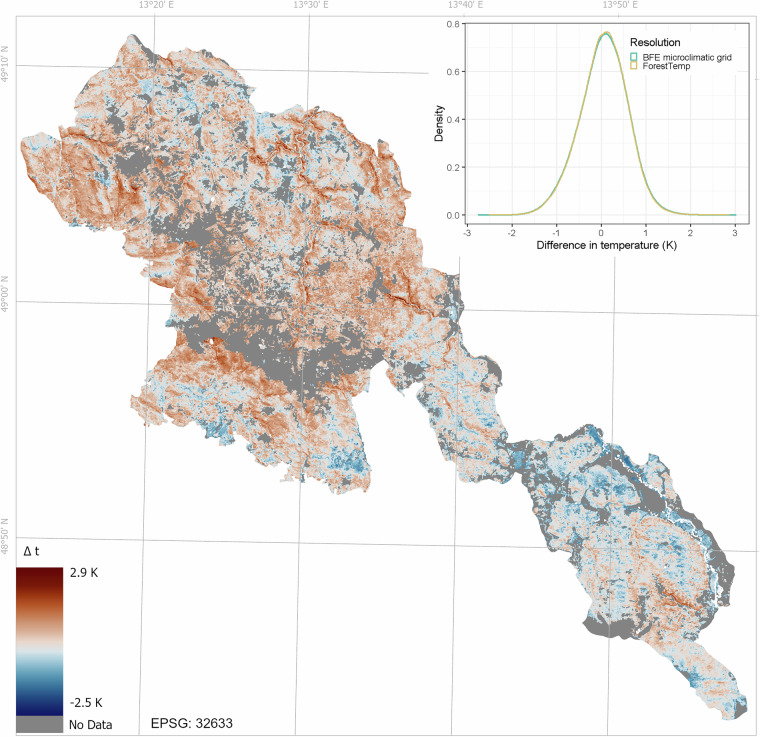
The difference in the annual mean near-ground temperature between the microclimatic grids for the Bohemian Forest Ecosystem and ForestTemp (BFE - ForestTemp). The negative values indicate cooler microclimatic temperatures in the BFE grids compared to ForestTemp. The map shows spatial patterns of temperature differences at the ForestTemp resolution (25 m), and the density plot shows the distribution of the differences at both the 5 m resolution of the microclimatic grids and at the 25 m resolution of ForestTemp. The numbers next to the zero-centred colour map represent the range of temperature differences between zonal means of the BFE microclimatic grids and ForestTemp observed within the Bohemian Forest Ecosystem.

For comparison, we also calculated the zonal mean of our mean annual near-ground temperature for each ForestTemp raster cell. To summarise the differences, we plotted the density distribution of the differences at both resolutions (Fig. 5). Overall, the BFE microclimatic grids provide near-ground temperature estimates similar to those of ForestTemp grids. The mean difference between the zonal mean of the BFE mean annual temperature grid and the ForestTemp mean annual temperature grid was 0.05 °C (BFE minus ForestTemp), indicating very similar near-ground temperatures (Table 5).

However, despite this small mean difference, we observed large spatial variation in local differences. These differences ranged from −2.51 °C to +2.88 °C for the 25 m grids and showed clear geographic patterns (Fig. 5). The BFE microclimatic grids show higher temperatures at higher elevations and lower temperatures at lower elevations compared with ForestTemp. Therefore, the main advantage of BFE grids compared to ForestTemp lies in their coverage of the disturbed forest areas and their better representation of the local – mostly topography driven – effects, for example, in deep and narrow valleys (Fig. 5).

#### Air temperature at 200 cm

Finally, we compared our high-resolution grids of minimum, mean, and maximum air temperatures and GDD at 200 cm with the ERA5-Land reanalysis data^75,76^. To downscale coarse-grain ERA5-Land grids with 9 km resolution to an ecologically more relevant resolution, we used regression kriging with elevation as a single model predictor and subsequent kriging of model residuals implemented in the KrigR R package^72^. The KrigR was developed specifically to downscale coarse-grain meteorological data such as ERA5-Land. We downscaled the ERA5-Land hourly data to the highest resolution recommended in the KrigR documentation, which corresponds to 0.02° (approximately 740 m at the study latitude). From these downscaled ERA5-Land datasets, we calculated the same set of microclimate variables as in our grids for the same time period.

**Fig. 6:**
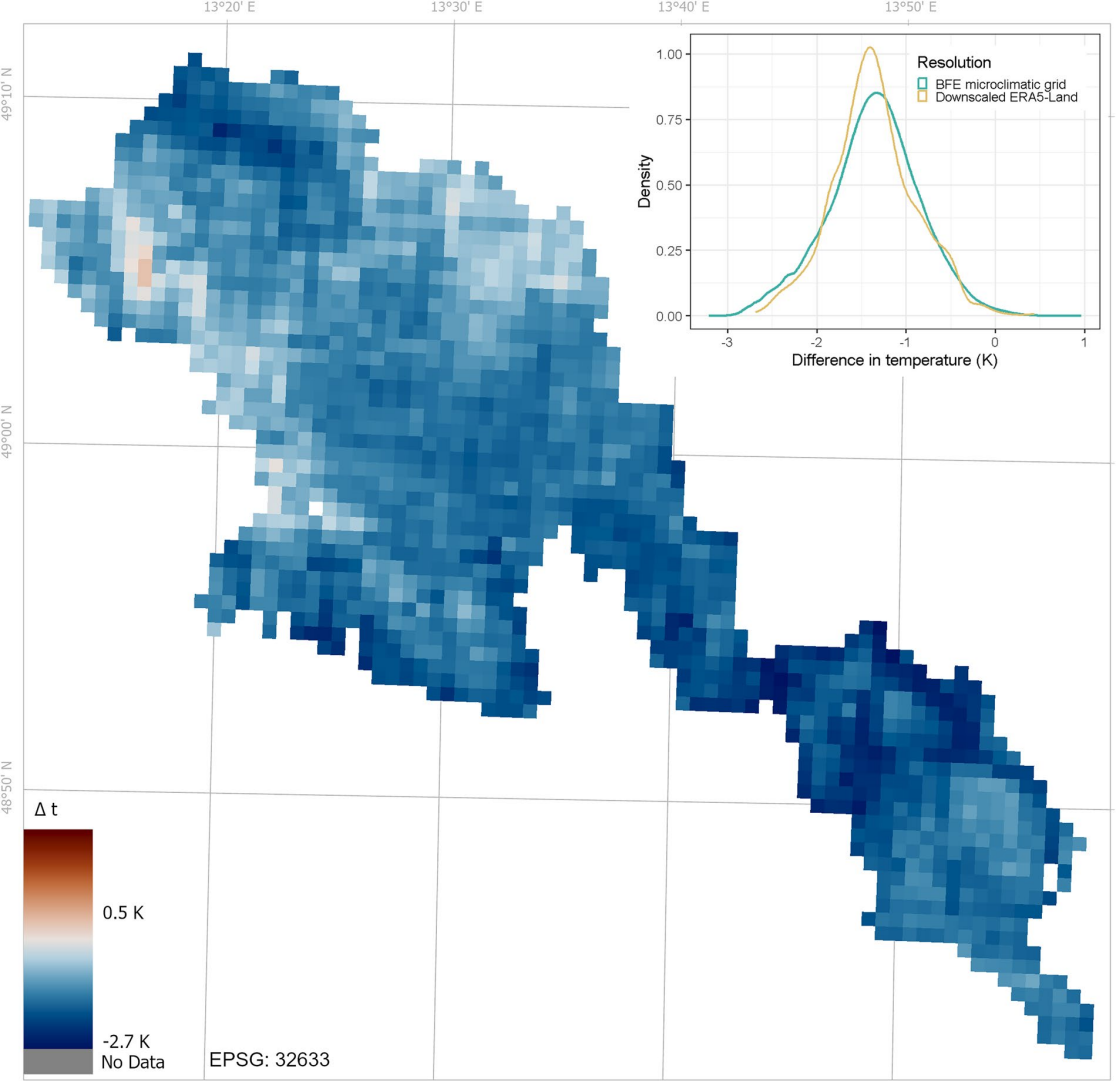
The difference in annual mean air temperature at 200 cm between the microclimatic grids for the Bohemian Forest Ecosystem and the downscaled ERA5-Land (BFE - downscaled ERA5-Land). The map shows spatial patterns of temperature differences at the resolution of the downscaled ERA5-Land (~740 m), and the density plot shows the distribution of the differences at both the 5 m resolution of the microclimatic grids and the downscaled ERA5-Land resolution. The numbers next to the zero-centred colour map represent the range of temperature differences between the zonal means of the BFE microclimatic grids and the downscaled ERA5-Land observed within the Bohemian Forest Ecosystem. Negative values indicate cooler temperatures in the BFE microclimatic grids compared to downscaled ERA5-Land.

For comparison, we also calculated the zonal mean of the BFE microclimatic grids at the 0.02° resolution of the downscaled ERA5-Land grid. Then we compared the BFE microclimatic grids with downscaled ERA5-Land data by calculating the difference between these datasets (BFE - ERA5-Land) in both the original 5 m resolution and upscaled 0.02° resolution (zonal mean). To summarise these differences, we plotted the density distribution of the differences at both resolutions (Fig. 6).

Compared to the downscaled ERA5-Land data, the BFE microclimatic grids showed lower mean annual air temperature at 200 cm (mean difference = −1.38 °C), lower 5^th^ percentile of minimum daily temperatures (mean difference = −2.29 °C), and lower growing degree days (mean difference = −293.66 °C d), while the 95^th^ percentile of maximum daily temperatures differed much less (mean difference = −0.3 °C; Table 5). This indicates strong topographic effects, especially on minimum temperatures driven by processes such as cold air pooling, which are not adequately captured in the ERA5-Land reanalysis data and are also not accounted for by the KrigR downscaling procedure.

The BFE microclimatic grids generally show much higher spatial variability in air temperature than the downscaled ERA5-Land, even when aggregated to the same resolution (Fig. 6). The temperatures extracted from BFE grids are also much closer to the temperatures measured at independent weather stations (Table 4), suggesting that ERA5-Land should be used for ecological applications in complex mountain terrain and forested landscapes with caution. Even after downscaling the ERA5-Land data through the KrigR R package, the resulting grids do not capture the temperature conditions experienced by organisms in our study region and show much reduced spatial variability. This can have important consequences for the identification of species refugia^77^ or the quantification of forest temperature buffering^7^.

These findings underscore the need to account for local topographic variability when using reanalysis datasets like ERA5-Land, particularly at finer scales where such variations can significantly impact results. Our analysis suggests that while downscaled ERA5-Land data provide a useful approximation, they underestimate temperature extremes, particularly cold extremes, compared to high-resolution local grids such as the BFE grids.

#### Comparison of measured temperatures with other grids

Using the data from our stations, we calculated the difference between each grid and our measured data (grid − logger). For the BFE grids, we used mean error (ME), mean absolute error (MAE), and RMSE from ten-fold spatial cross-validation, which provides an independent estimate comparable to the grid–station errors reported for the other products (Table 6).

**Table 6:** Comparison of available products with values of the same variable from our loggers. The soil and air temperatures at 15 cm were compared at 168 sites for SoilTemp and 122 for ForestTemp. The air temperatures at 200 cm were compared at 262 sites. ME, MAE, and RMSE were calculated as bilinearly interpolated grid value minus logger value (grid − logger).

Variable (unit)	Data source	Resolution	ME	MAE	RMSE
T.soil_8_cm.mean (°C)	SoilTemp	1000 m	−2.68	2.69	2.86
	BFE microclimatic grid	5 m	0.01	0.32	0.41
T.air_15_cm.mean (°C)	ForestTemp	25 m	−0.11	0.46	0.57
	BFE microclimatic grid	5 m	0.007	0.371	0.46
T.air_200_cm.mean (°C)	Downscaled ERA5-Land	740 m	1.36	1.37	1.49
	ERA5-Land	9 000 m	1.59	1.59	1.73
	BFE microclimatic grid	5 m	−0.003	0.33	0.42

## Usage Notes

**Fig. 7:**
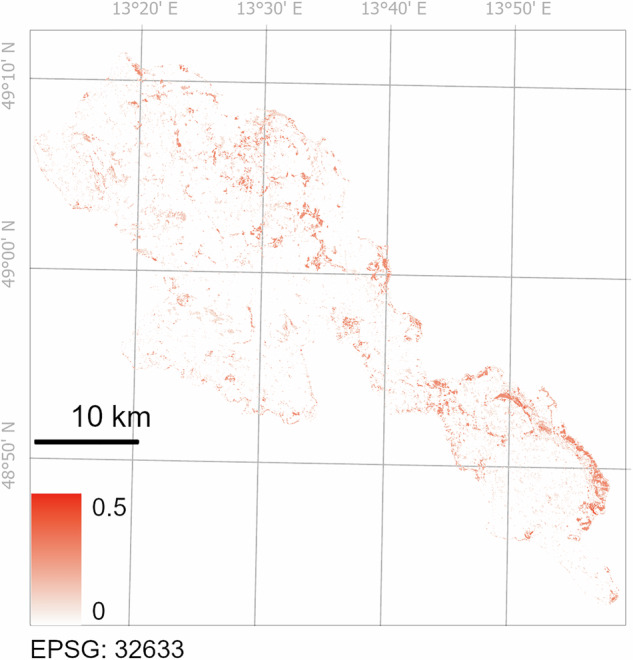
The uncertainty raster shows, for each pixel within the area, the proportion of variables for which our microclimatic models extrapolate beyond the variable range covered by the training dataset.

Although our microclimatic network covers the main environmental gradients in the Bohemian Forest Ecosystem, areas with extreme slopes, the highest diurnal anisotropic heating, and the tallest trees have limited coverage (Table 2). In these cases, our models extrapolate, and the predictions for these areas should be interpreted with caution. We produced an uncertainty raster (Fig. 7) by assessing the extrapolation of predictor variables. For each predictor, we classified raster cells outside the range of that predictor covered by our microclimatic loggers as 1 (extrapolated) and those within the range as 0. We summed the binary rasters of each predictor and divided the resulting sum by the number of predictors to create a raster showing the uncertainty of the microclimatic predictions. The resulting uncertainty raster thus shows, for each pixel within the area, the proportion of predictors for which our microclimatic models extrapolate beyond the predictor range covered by the training dataset. Higher values correspond to areas with higher uncertainty.

In both National Parks, we deliberately focused only on the forested area (80% of the entire area). The sites covered the full range of canopy cover – from mature closed canopy forests to completely treeless forest stands at recently disturbed sites. Therefore, while the BFE microclimatic grids correctly reflect conditions in treeless forest stands after large-scale disturbances, it is important to be cautious when using them in areas outside forested regions (e.g. built-up areas, wet meadows, and peatlands).

Our models predict air temperatures at 200 cm more accurately than near-ground air temperatures. Temperatures near the ground are generally more heterogeneous and therefore more challenging to model^12^ due to factors such as surface roughness, vegetation density, and soil conditions. In the future, models of near-ground temperature could potentially be improved by including predictors that capture the effects of understorey vegetation^6,78^, or local soil moisture dynamics^51,79^, or direct solar radiation.

Finally, while our dataset spans a single year, the maps generated from this study provide novel insights into temperature heterogeneity across the BFE. To support this use, we tested the correlation of all microclimate variables for the modelled year and the next year at all our sites that were active in both years. Across all microclimate variables, site-level rankings were highly consistent between years (Spearman ρ ≈ 0.94–0.99) except near-ground minima (T.air_15_cm.min.5p; Spearman ρ ≈ 0.59), indicating stronger year-to-year variability in near-ground minimum temperatures. This pattern is influenced by the duration of snow cover in the area, which has high interannual variability.

To extend these findings over longer periods, we recommend generating annual maps by adjusting predictions with offsets from local climate stations and incporporating time-vayring information on forest structure. 

## Supplementary information

Supplementary figures S01 - S10

## Data Availability

High-resolution microclimatic grids for the Bohemian Forest Ecosystem are freely available in the Zenodo repository: https://zenodo.org/records/17671120 with 10.5281/zenodo.17671120^71^.

## Code availability

The code for modelling is available in the Zenodo repository under DOI: 10.5281/zenodo.17671120^71^, the same repository as the grids. It includes microclimatic variables, topography and forest structure variables for all stations used for modelling.

The LiDAR data can be obtained from the Administration of Bavarian Forest National Park and the Šumava National Park Administration. The LiDAR-derived topography, and forest structure rasters used for grid predictions are not publicly available; however, they can be obtained upon request from the authors. The code also includes inactive parts that demonstrate the procedures used for interpolating and generating uncertainty maps, which rely on the unpublished rasters.
