# Supplementary material for: High-resolution microclimatic grids for the Bohemian Forest Ecosystem based on in situ measurements
Source: Sci Data. 2026 Jan 14;13:246. doi: 10.1038/s41597-026-06566-z (PMC12913920; doi:10.1038/s41597-026-06566-z)

## **Fig. S01 – S09** Partial dependence plots showing partial effects of each predictor variable selected in particular model. Rug along the x-axis show distribution of training data points. Y-axis labels (panel titles for interaction terms) combine predictor name and empirical degrees of freedom of the smooth term with higher number indicating more complex shapes; s() stands for univariate smooths, te() for full tensor product smooth, shaded area is 95% confidence interval.

## **Fig. S01** T.soil_8_cm.mean


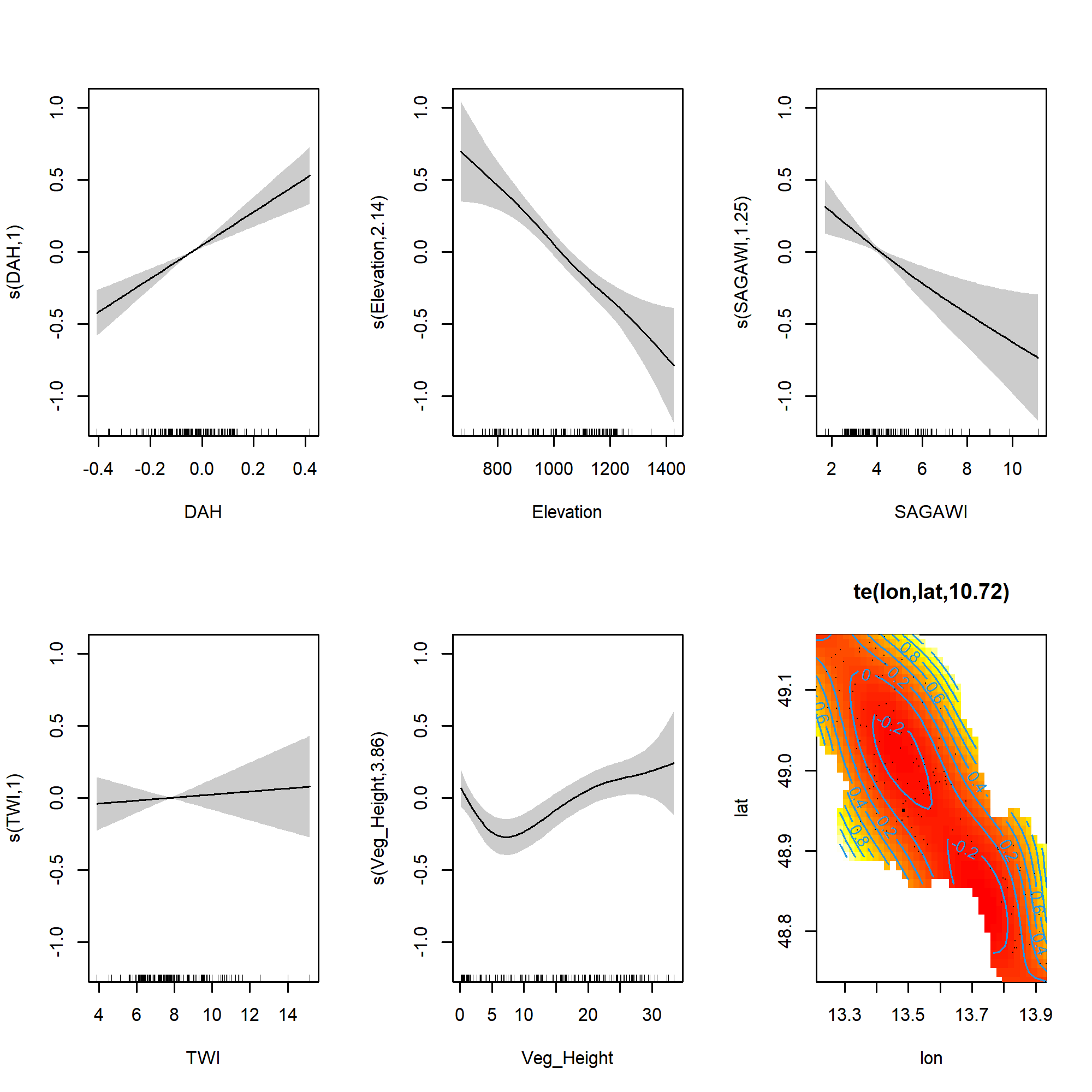


## **Fig. S02** spatial_T.air_15_cm.mean


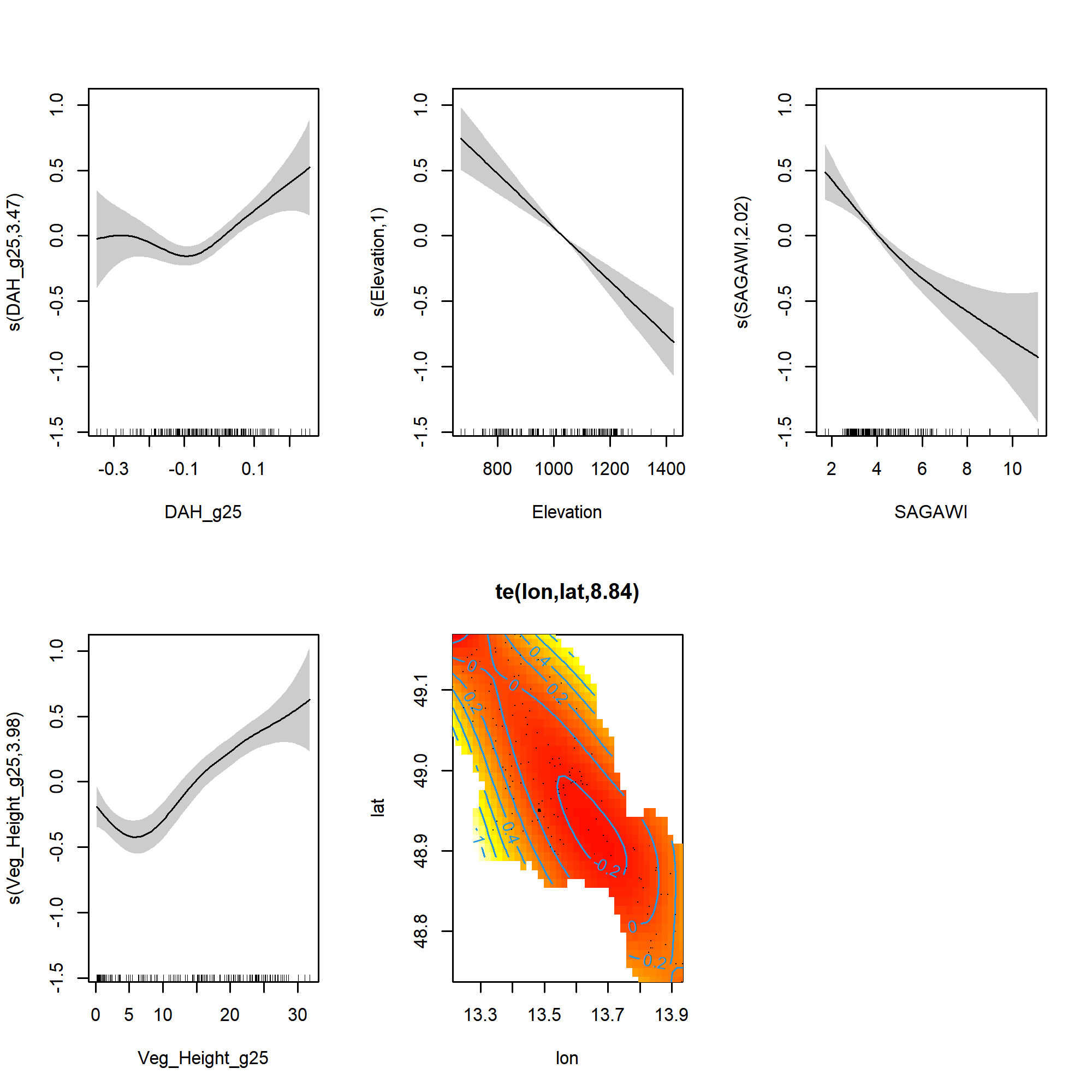


## **Fig. S03** T.air_15_cm.max.95p


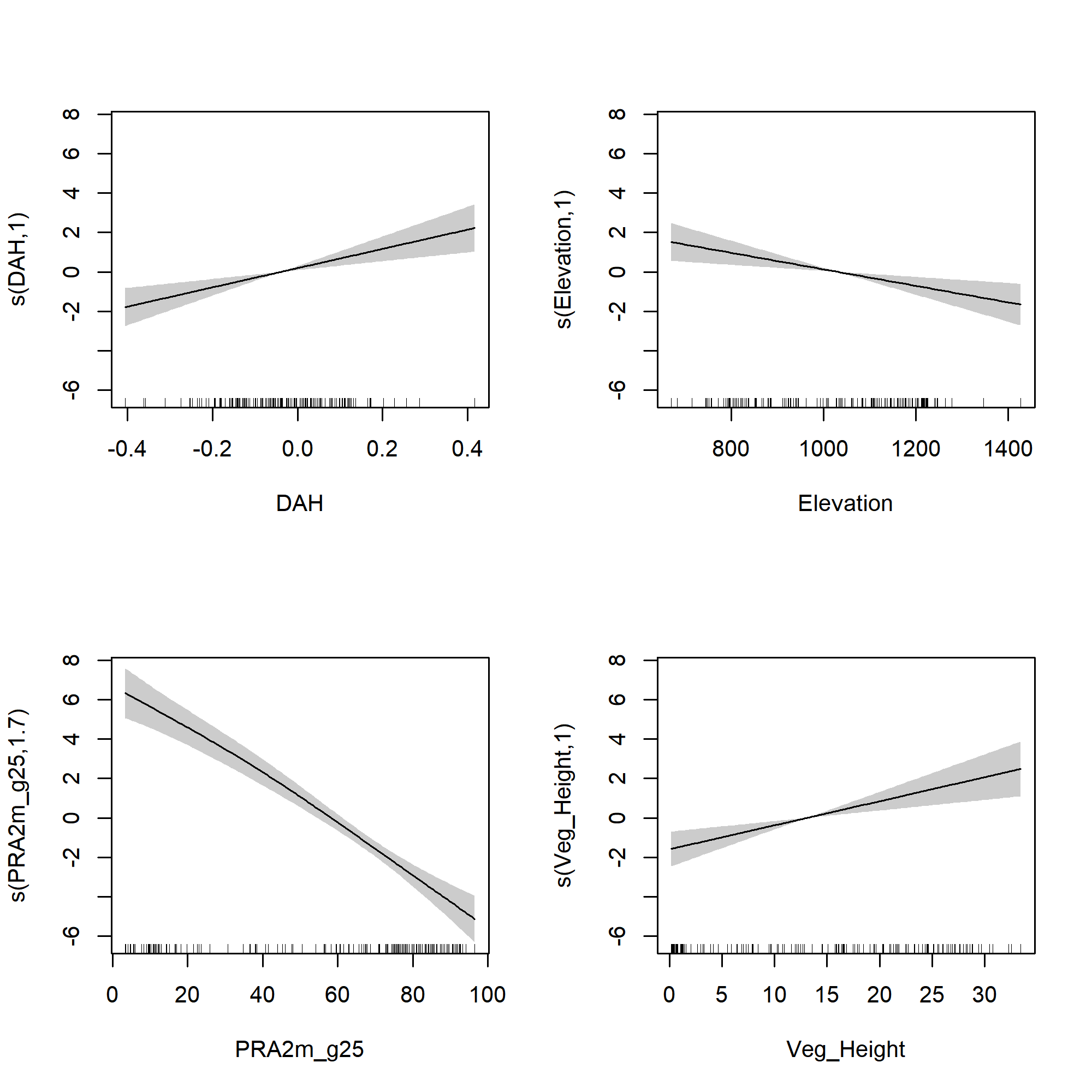


## **Fig. S04** T.air_15_cm.min.5p


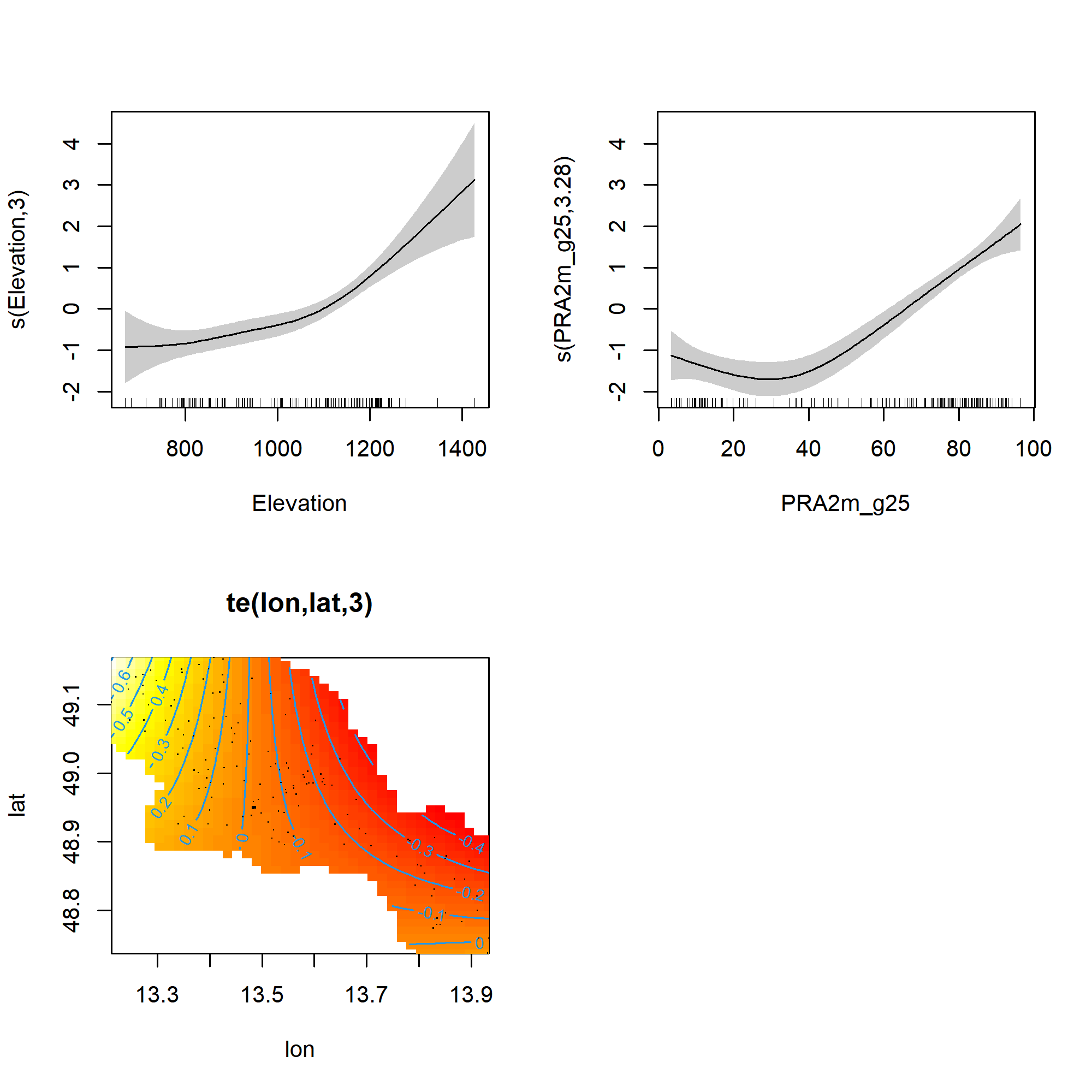


## **Fig. S05** T.air_15_cm.GDD5


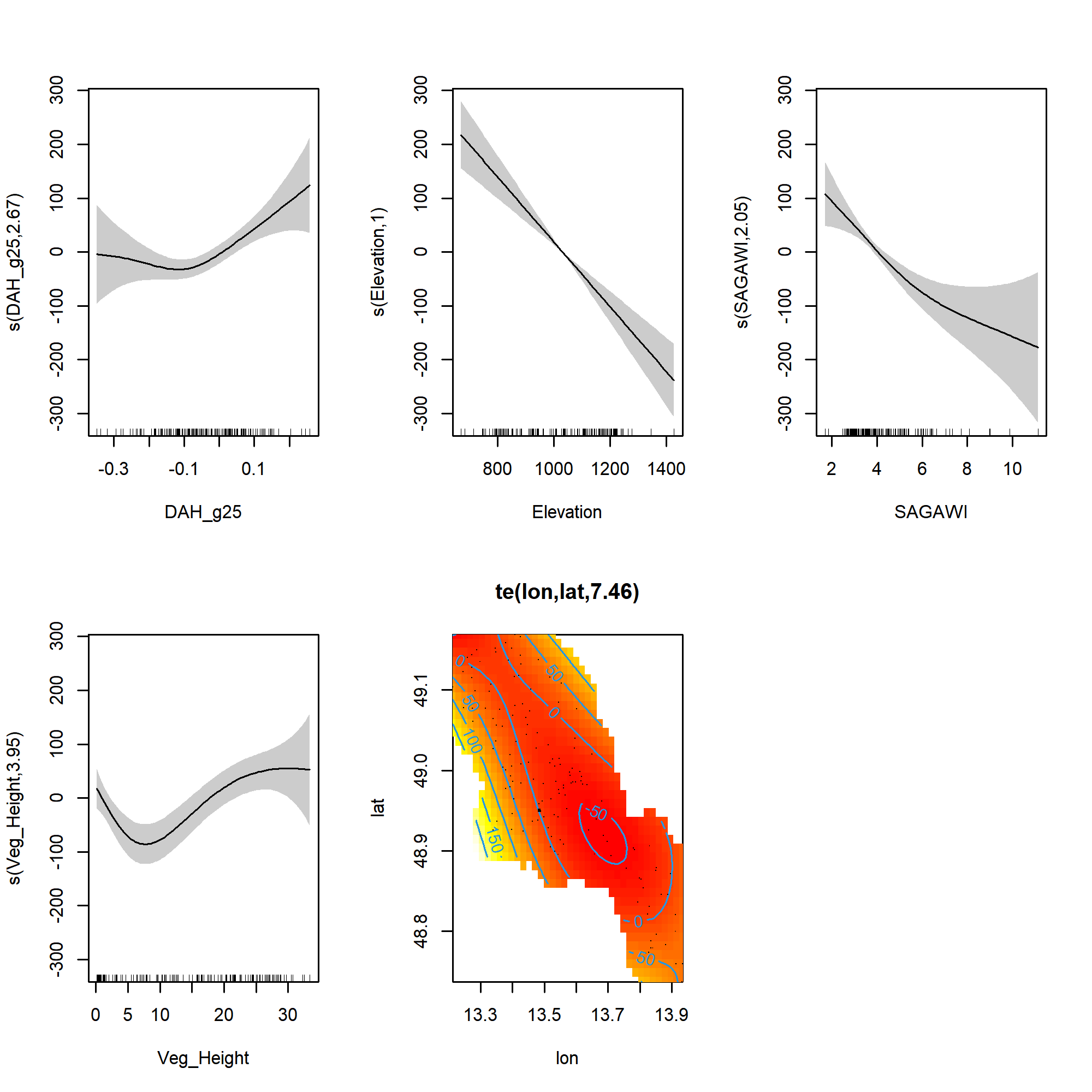


## **Fig. S06** T.air_200_cm.mean


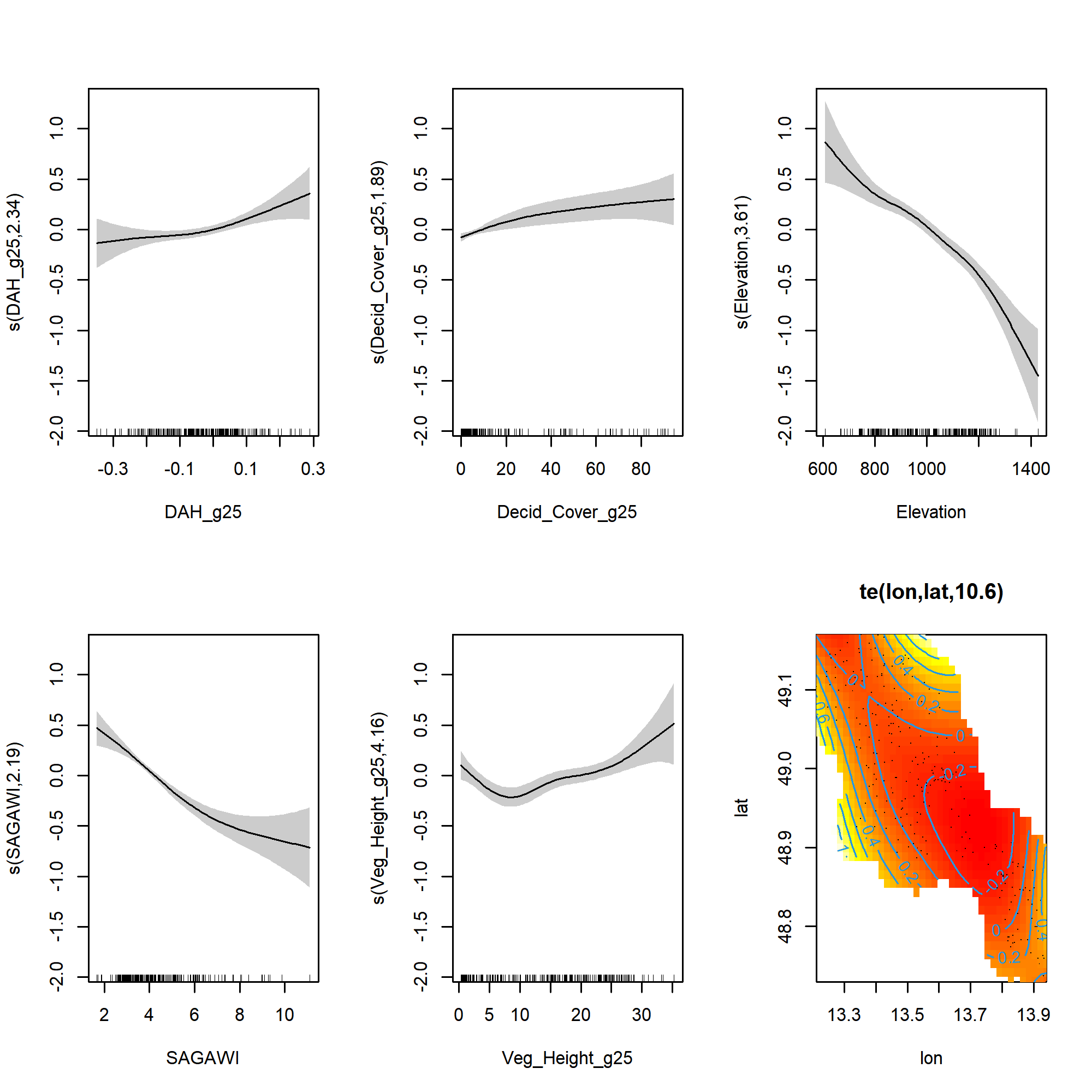


## **Fig. S07** T.air_200_cm.max.95p


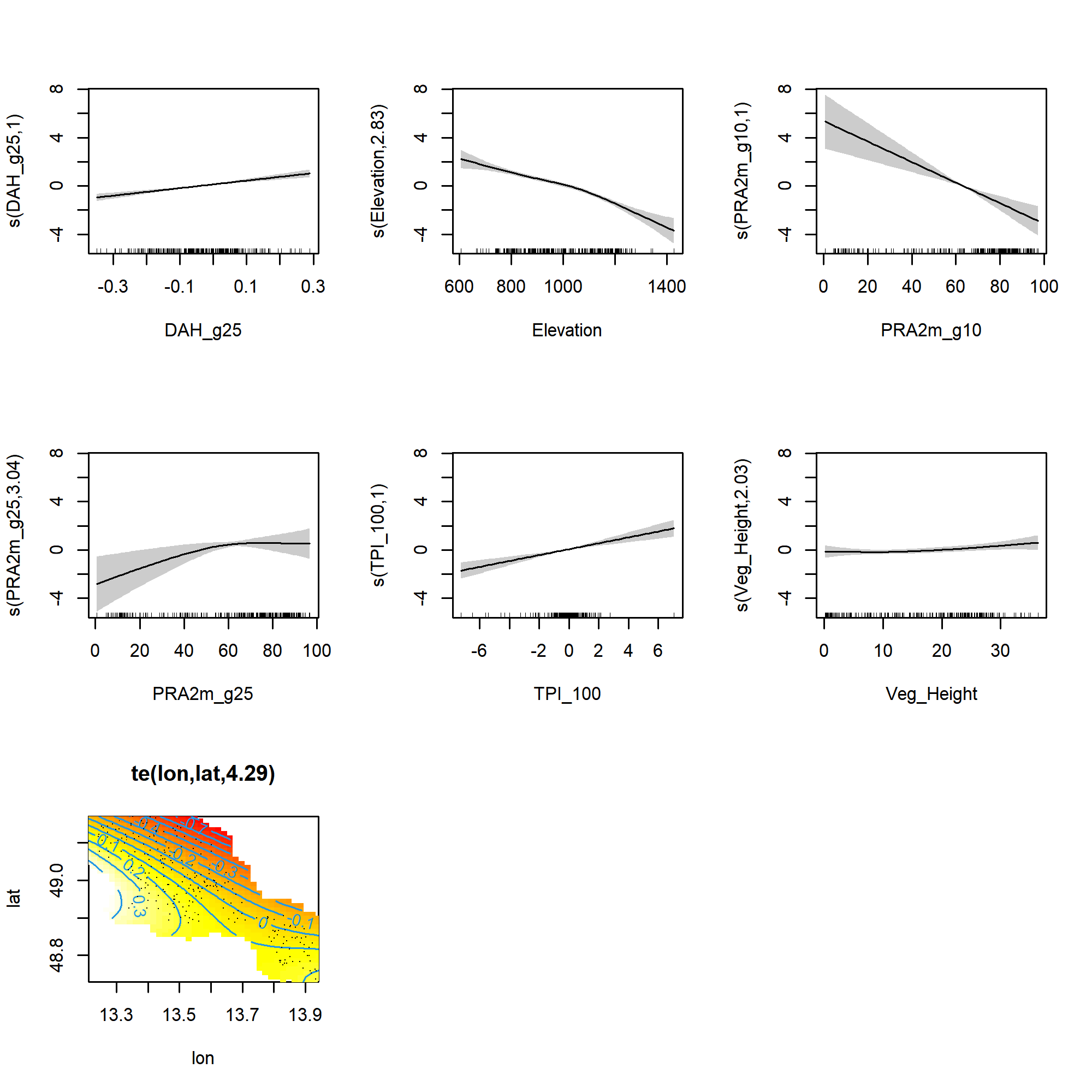


## **Fig. S08** T.air_200_cm.min.5p


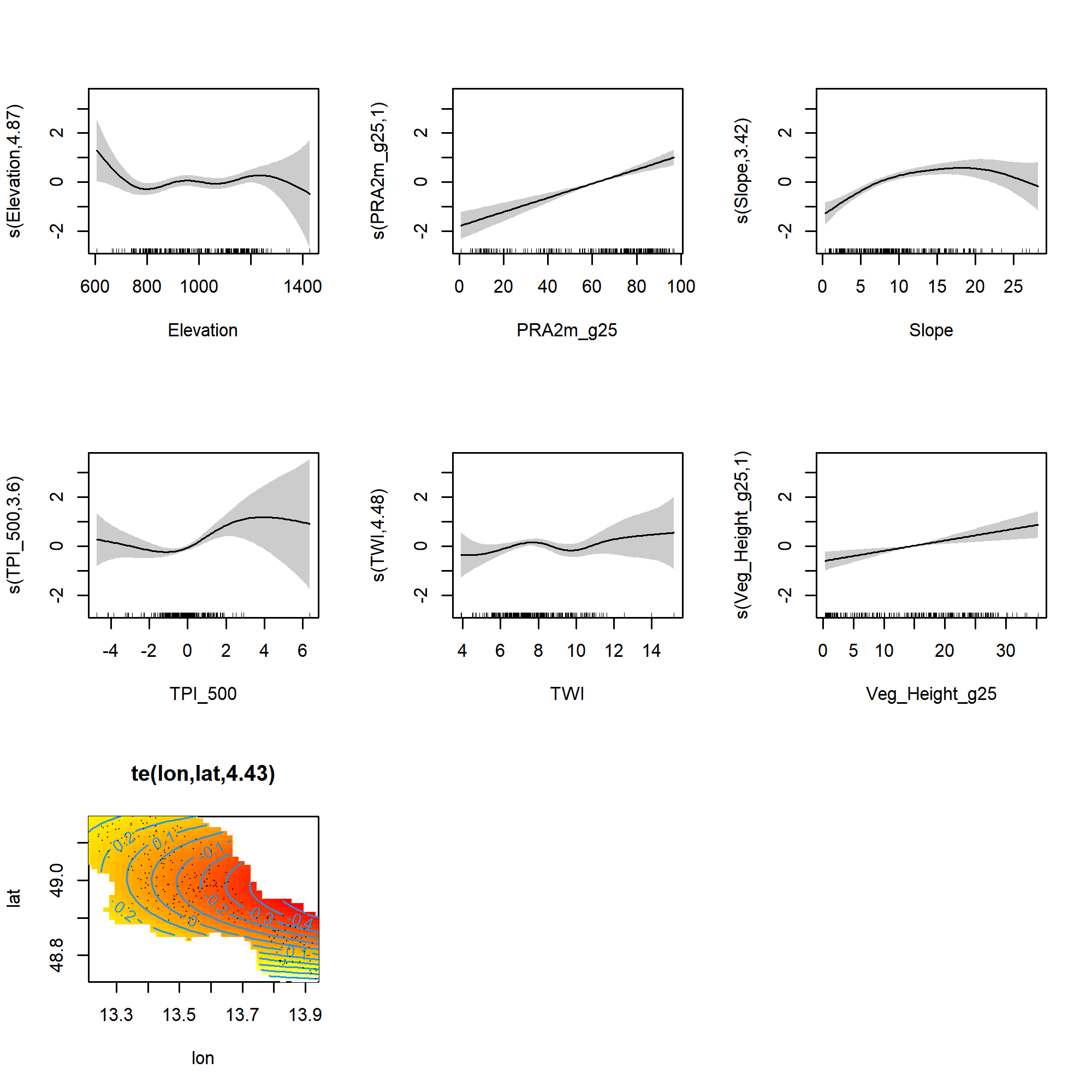


## **Fig. S09** T.air_200_cm.GDD5


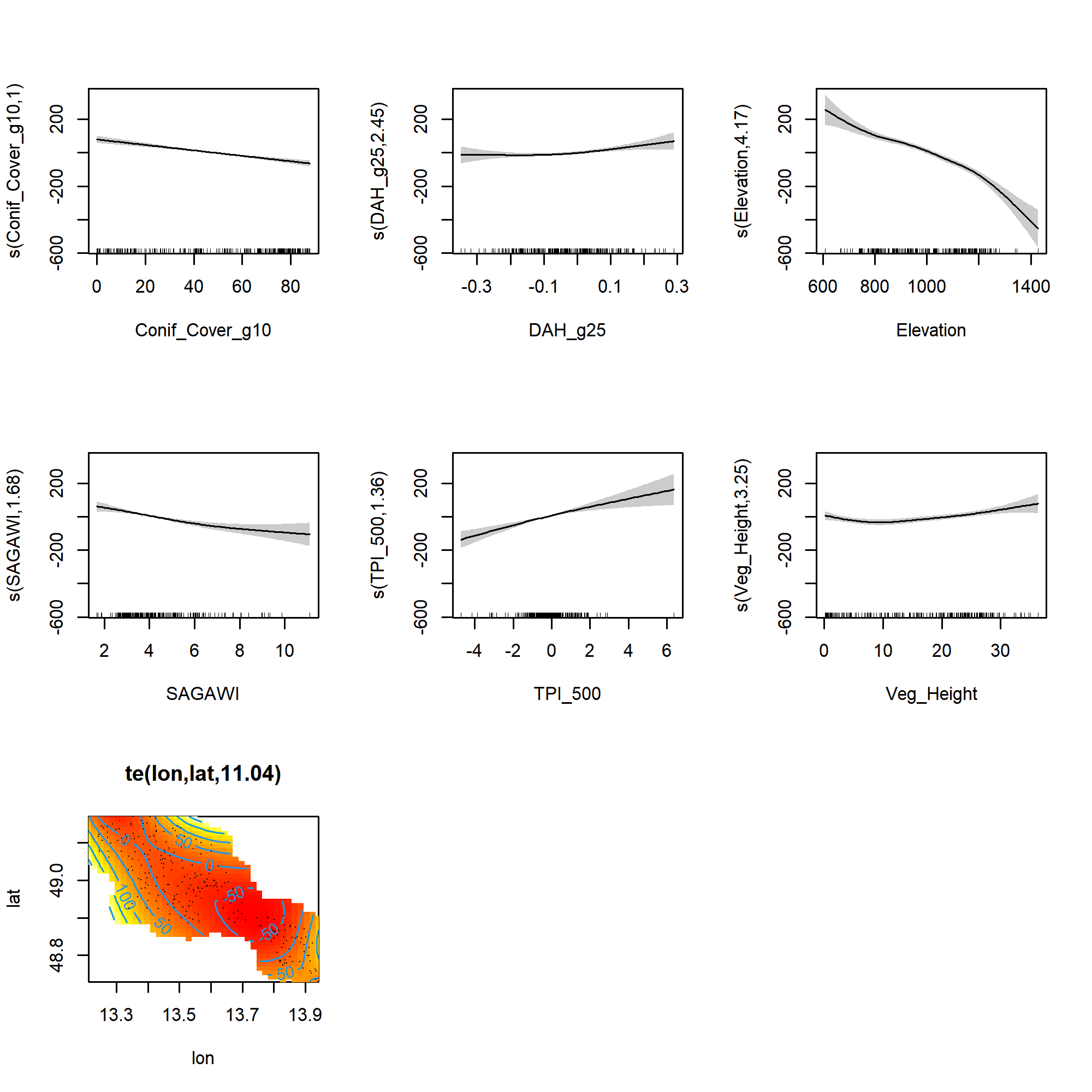


## **Fig. S10** Coverage of environmental gradients in BFE vs. covered by our sites
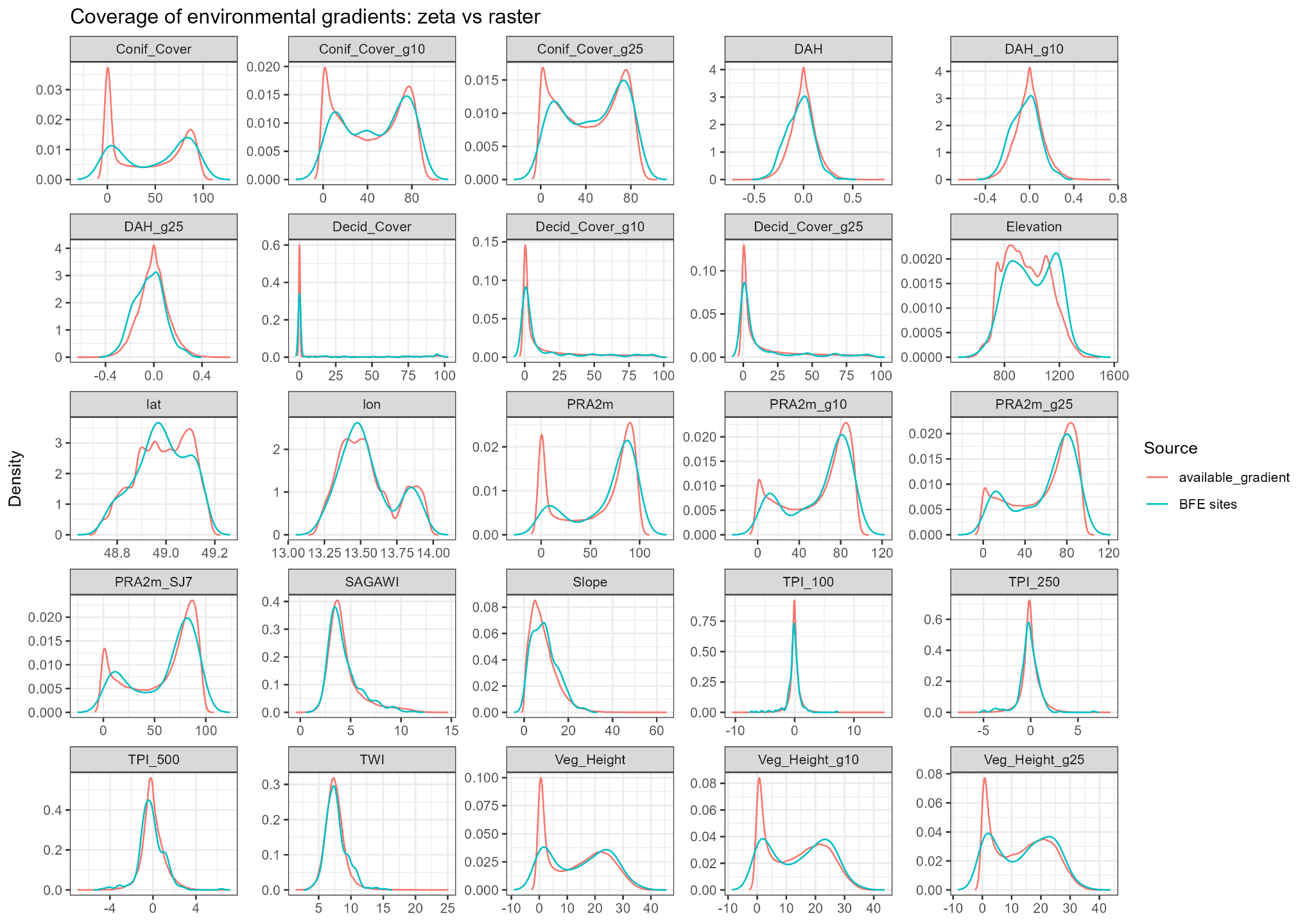

Supplement: Supplementary file 1 — Supplementary figures S01 - S10 [file 41597_2026_6566_MOESM1_ESM.docx]
